# PCSK9 increases vulnerability of carotid plaque by promoting mitochondrial dysfunction and apoptosis of vascular smooth muscle cells

**DOI:** 10.1111/cns.14640

**Published:** 2024-02-25

**Authors:** Ran Xu, Tianhua Li, Jichang Luo, Xiao Zhang, Tao Wang, Yabing Wang, Yan Ma, Bin Yang, Jinzhu Jia, Adam A. Dmytriw, Wenjing Li, Liqun Jiao

**Affiliations:** ^1^ Department of Neurosurgery, Xuanwu Hospital Capital Medical University Beijing China; ^2^ China International Neuroscience Institute (China‐INI) Beijing China; ^3^ School of Public Health and Center for Statistical Science Peking University Beijing China; ^4^ Neuroendovascular Program, Massachusetts General Hospital Harvard Medical School Boston Massachusetts USA; ^5^ Laboratory of Computational Biology and Machine Intelligence, National Laboratory of Pattern Recognition Institute of Automation, Chinese Academy of Sciences Beijing China; ^6^ School of Artificial Intelligence University of Chinese Academy of Sciences Beijing China; ^7^ Department of Interventional Neuroradiology, Xuanwu Hospital Capital Medical University Beijing China; ^8^ Daepartment of Neurosurgery and Neurology, Jinan Hospital of Xuanwu Hospital Shandong First Medical University Jinan China

**Keywords:** apoptosis, carotid plaque, mitochondria dysfunction, PCSK9, vascular smooth muscle cells

## Abstract

**Background:**

Proprotein convertase subtilisin/kexin type 9 (PCSK9) has been recognized as a novel lipid‐lowing target. Recent clinical studies suggested the value of inhibiting PCSK9 in decreasing the vulnerability of coronary plaques. However, the evidence of PCSK9‐regulated evolution of unstable carotid plaques is unclear, which has limited the use of PCSK9 inhibitor in carotid plaques. This study aimed to determine the effect and molecular mechanisms of PCSK9 on vulnerability of carotid plaques, to provide potential therapeutic targets for stabilizing carotid plaques.

**Methods:**

The expression of PCSK9 in stable and unstable carotid plaques were examined in tissue and plasma. Human aortic vascular smooth muscle cells (VSMCs) and carotid VSMCs were employed to transfect lentivirus for overexpression and knockdown of PCSK9, respectively. Morphological and functional changes of mitochondria were observed by live‐cell imaging. Cell apoptosis was evaluated by propidium iodide staining. RNA‐sequencing and biological examinations were performed to explore and validate the underlying mechanisms. Truncated plasmids were employed to identify the functional domain of PCSK9 in regulation of VSMCs' mitochondrial morphology, function and apoptosis.

**Results:**

Clinically, PCSK9 was closely related with vulnerability of human carotid plaques. Increased expression of PCSK9 in human VSMCs was accompanied by higher level of apoptosis. At subcellular level of VSMCs, the morphology of mitochondria was shifted toward the fission state, followed by mitochondrial dysfunction. Inhibition of p38 MAPK activation partially rescued the above morphological and behavioral changes caused by PCSK9. Furthermore, inhibiting of dynamin‐related protein 1 (DRP1) attenuated PCSK9‐related mitochondrial dysfunction and cell apoptosis. The 1‐149aa domain of PCSK9 protein was essential to achieve functional regulation to VSMCs.

**Conclusion:**

Our findings demonstrated that PCSK9 induced morphology‐related mitochondrial dysfunction and apoptosis of VSMCs, which may be related to increased vulnerability of carotid plaque.

## Graphical abstract

PCSK9 induces mitochondrial dysfunction and apoptosis of vascular smooth muscle cells through p38‐DRP1 pathway, which contributes to development of carotid vulnerable plaque. We provided theoretical evidence to explore the application of PCSK9 inhibitor to prevent carotid plaque rupture, so as to decrease the incidence of ischemic stroke.

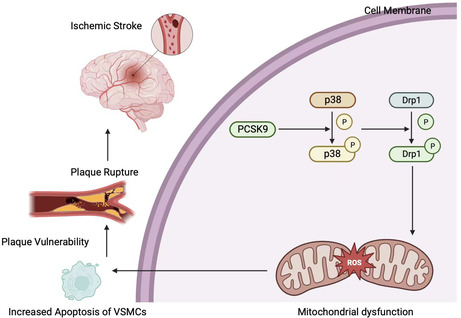

## 1 INTRODUCTION

Ischemic stroke is one of the main causes of death and disability in the world, and its incidence is increasing year by year in developing countries, causing a heavy burden.^1^, ^2^ Carotid atherosclerotic stenosis (CAS) accounts for 15%–20% of the causes of ischemic stroke,^3^ especially in the Western Pacific region.^4^ Carotid endarterectomy can benefit symptomatic CAS patients with luminal stenosis of 70%–99%, but the benefit in symptomatic CAS patients with stenosis less than 70% and asymptomatic CAS patients remains unclear.^5^, ^6^ Compared with treatment decisions based on luminal stenosis, screening of unstable plaque according to structural evaluation was more beneficial to stroke risk prediction and surgical population selection in CAS patients.^7^ The characterized pathological features of unstable plaques include thin fibrous caps, large lipid cores, abundant infiltration of inflammatory cells, and intraplaque hemorrhage. The current evaluation of unstable carotid plaque was mainly based on imaging, and the accuracy was not sufficient to meet clinical practice.^8^ In addition, research on peripheral blood biomarkers of unstable plaques is only at the preliminary stage of exploration. Discovery of unstable plaque biomarkers, screening of high‐risk CAS populations, making individualized diagnosis and treatment will reduce the incidence of ischemic events.

Vascular smooth muscle cells (VSMCs) are the key cell type to maintain vasomotor and pathophysiological functions. In stable plaques, VSMCs secrete extracellular matrix (ECM) to construct plaque fibrous caps. However, in unstable plaques, the apoptosis of VSMCs are abnormally increased, and the incidence of plaque rupture and thromboembolism is higher accordingly.^9^ Previous studies have shown that mitochondrial dysfunction can promote VSMCs' apoptosis, but the exact molecular mechanism remains unclear.^10^ Furthermore, the association between this phenomenon and CAS plaque vulnerability remains to be confirmed.

The mutation of proprotein convertase subtilisin/kexin type 9 (PCSK9) was first discovered in a family with autosomal dominant familial hypercholesterolemia. Gain‐of‐function mutation of PCSK9 can significantly increase the protein level of low‐density lipoprotein cholesterol (LDL‐C). PCSK9 can bind and accelerate the lysosomal degradation of low density lipoprotein receptor (LDLR) on the cell surface, thereby inhibiting the uptake of LDL‐C by liver cells, resulting in the increase of LDL‐C in plasma and accelerating the progression of atherosclerosis.^11^ The inhibitors of PCSK9 have been widely recognized in the treatment of atherosclerosis regarding to lipid metabolism. Clinical trials such as FOURIER and ODESSEY, showed that both evolocumab and alirocumab could reduce the incidence of ischemic events in patients with high‐risk coronary heart disease.^12^, ^13^ Using with near‐infrared light and intravascular ultrasound, Hiroyuki et al found that PCSK9 inhibitors have a stronger effect on stabilizing coronary plaque than statins.^14^ PCSK9 inhibitors combined with rosuvastatin can also thicken the fibrous cap, reduce the lipid angle and lower the lipid load index, thereby stabilizing high‐risk plaques.^15^ In summary, in addition to the lipid‐lowering effect, the clinical value of PCSK9 in stabilizing plaque has been preliminarily confirmed in coronary arteries. However, the potential effect of PCSK9 in stabilizing carotid plaques and the specific pathway remains to be investigated.

The aim of the current study was to explore the regulation and underlying mechanisms of PCSK9 on carotid plaques from the perspective of VSMCs' mitochondrial dysfunction and apoptosis. The clinical association between PCSK9 and vulnerability of carotid plaques was also be evaluated. We sought to provide clues and evidence for stabilizing carotid plaques and decreasing the incidence of ischemic stroke, so as to expand the application of PCSK9 inhibitors.

## 2 MATERIALS AND METHODS

### 2.1 Specimen collection

Carotid plaques and blood plasma were obtained from patients who received carotid endarterectomy (stenotic extent >50% for symptomatic patients and >70% for asymptomatic patients according to the North American Symptomatic Carotid Endarterectomy Trial criteria for carotid stenosis)^16^ at Xuanwu Hospital, Capital Medical University. Plaques were classified into stable and unstable types based on ultrasound and histopathological findings. Unstable plaques are characterized by intraplaque hemorrhage, mural thrombus, thin fibrous caps, or an incomplete fibrous cap, otherwise they are known as stable plaques.^17^

### 2.2 Cell culture and treatment

Human carotid VSMCs (HC‐VSMCs) and human aortic VSMCs (HA‐VSMCs) were obtained from Bluefbio (Shanghai, China), cultured in Dulbecco modified eagle medium (DMEM, Life Technologies, USA), supplemented with 10% fetal bovine serum (FBS, Thermo Fisher Scientific, USA), and cultured in 5% CO_2_ incubator at 37°C. Lentivirus with knockdown or overexpression of human PCSK9 was constructed by GeneChem (Shanghai, China). The sequences of lentivirus were listed as follows: negative control (NC) 5′‐TTCTCCGAACGTGTCACGT‐3′; sh‐PCSK9 5′‐ GGAAGAGACCCAGGAGGATAA‐3′. The plasmid for truncated mutant of human PCSK9 (1‐149aa, 155‐461aa, 450‐692aa) was designed according to the protein domains of PCSK9 in https://www.uniprot.org and synthesized by OBiO Technology (Shanghai, China). Transient transfections for plasmids were performed using Lipofectamine 3000 (Thermo Fisher Scientific). After infection for 2 days, VSMCs were cultured in DMEM containing 2 μg/mL puromycin (Thermo Fisher Scientific) for an additional 2 weeks. Mdivi‐1, an inhibitor of DRP1 (Med Chem Express, China), was dissolved in DMSO (working concentration: 1 μM) before application. The small molecule SB203580 (Selleck, China) was dissolved in DMSO (working concentration: 10 μM) and used as an inhibitor of p38 phosphorylation.

### 2.3 RNA‐sequencing and bioinformatics analyses

Total RNA from control and sh‐PCSK9 VSMCs was isolated for the mRNA sequencing library. Briefly, after RNA quality control and purification and cDNA synthesis, a 300–400 bp library was prepared. The established library was submitted to quality control by Agilent 2100 Bioanalyzer, and finally sequenced using the Illumina next‐generation sequencing platform according to the manufacturer's instructions. mRNA with a |log2FoldChange|>1 and *p* < 0.05 was considered differentially expressed. Database for annotation, visualization, functional annotation and the KEGG pathway analysis were adopted to obtain target gene‐enriched pathways. *p* < 0.05 was the criterion for statistical significance.

### 2.4 Reverse‐transcription polymerase chain reaction (RT‐PCR)

Total RNA was isolated from VSMCs using Trizol reagent (Takara, Tokyo, Japan) strictly according to instructions. cDNA was synthesized from total RNA (2 μg) through reverse transcription kit (Toyobo Life Science, Shanghai, China). Quantitative PCR (qPCR) was performed using 1 μL of cDNA in a standard SYBR premix Ex Taq (Takara) in the Roche LightCycler® 480 System (Roche, Indianapolis, IN, USA). The mRNA level was then normalized to the level of GAPDH mRNA and the relative expression of each mRNA was calculated using the 2^−ΔΔCt^ method. The following primers were used: GAPDH, 5′‐AATGAAGGGGTCATTGATGG‐3′, 5′‐AAGGTGAAGGTCGGAGTCAA‐3′; PCSK9, 5′‐CCTGGAGCGGATTACCCCT‐3′, 5′‐CTGTATGCTGGTGTCTAGGAGA‐3′.

### 2.5 Enzyme‐linked immunosorbent assay (ELISA)

The plasma from patients with CAS was added to an enzyme‐linked plate (Abcam, Cambridge, UK) and incubated overnight at 4°C. After incubation with goat serum, antibody of PCSK9 was added and incubated at 37°C for 1 h. Then, the secondary antibody was added and incubated at 37°C for 1 h. 3, 3′,5,5′‐Tetramethylbenzidine (TMB) solution was then added and kept at 25°C for 3 min. Terminating the reaction by adding 2 M H_2_SO_4_ solution. The absorbance at 450 nm was obtained after 5 min.

### 2.6 Histopathological examination

Carotid plaques were fixed with 4% paraformaldehyde, dehydrated with gradient ethanol, transparent with xylene, embedded in paraffin, and then fixed in a tissue slicer (thickness: 4 μm). Fixed tissue was stained with hematoxylin–eosin (H&E), masson according to standard protocol. Immunohistochemical (IHC) staining were performed using antibody of PCSK9 (Cell Signaling Technology, Beverly, MA, USA) according to protocol as previously reported.^18^

### 2.7 Live‐cell imaging

Mitochondria were stained with Mitotracker (Thermo Fisher Scientific) at a ratio of 1:5000 at 37°C for 10 min. Nuclei were stained with Hoechst33342 (Cell Signaling Technology) and incubated at 37°C for 20 min. The images achieved under confocal microscopy (Nikon, TiE) was analyzed by Mitochondria Analyzer in Image J. Reactive Oxygen Species (ROS) was determined by 10 μM dihydroethidium (DHE, Thermo Fisher Scientific) staining at 37°C for 10 min. Membrane potential was determined by incubating cells with 100 nM tetramethylrhodamine ethyl ester (TMRE, Thermo Fisher Scientific) at 37°C for 10 min. Confocal microscopy was used for image capture followed by analysis with Image J.

### 2.8 Evaluation of cell apoptosis

Apoptotic cells were stained with propidium iodide (PI) staining solution (Cell Signaling Technology), and nuclei was stained with Hoechst33342 (Cell Signaling Technology). Cells were maintained at 37°C with 5% CO_2_ in an on‐stage incubator (Tokai Hit, USA) during imaging. Five fields of views from each well were randomly selected and counted with Image J software to determine the proportion of apoptotic cells.

### 2.9 Immunofluorescence and immunoblotting

Immunofluorescence and immunoblotting were performed as previously described.^19^ The following antibodies were used: PCSK9, GAPDH, β‐actin, β‐Tubulin, Bcl2, Caspase‐3, Cleaved Caspase‐3, p38, p‐p38 (Cell Signaling Technology).

### 2.10 Statistical analysis

Data are presented as the mean ± the SD. Continuous variables were tested for normal distribution using the nonparametric one‐sample Kolmogorov–Smirnov test. The independent samples *t*‐test was used to test for normally distributed continuous variables. Data that do not exhibit a normal/Gaussian distribution were analyzed via a non‐parametric equivalent. Statistical analysis was conducted using GraphPad Prism version 9.00 software (GraphPad). *p* values <0.05 were considered to be statistically significant.

## 3 RESULTS

### 3.1 PCSK9 expression is increased in patients with unstable carotid plaques

**FIGURE 1:**
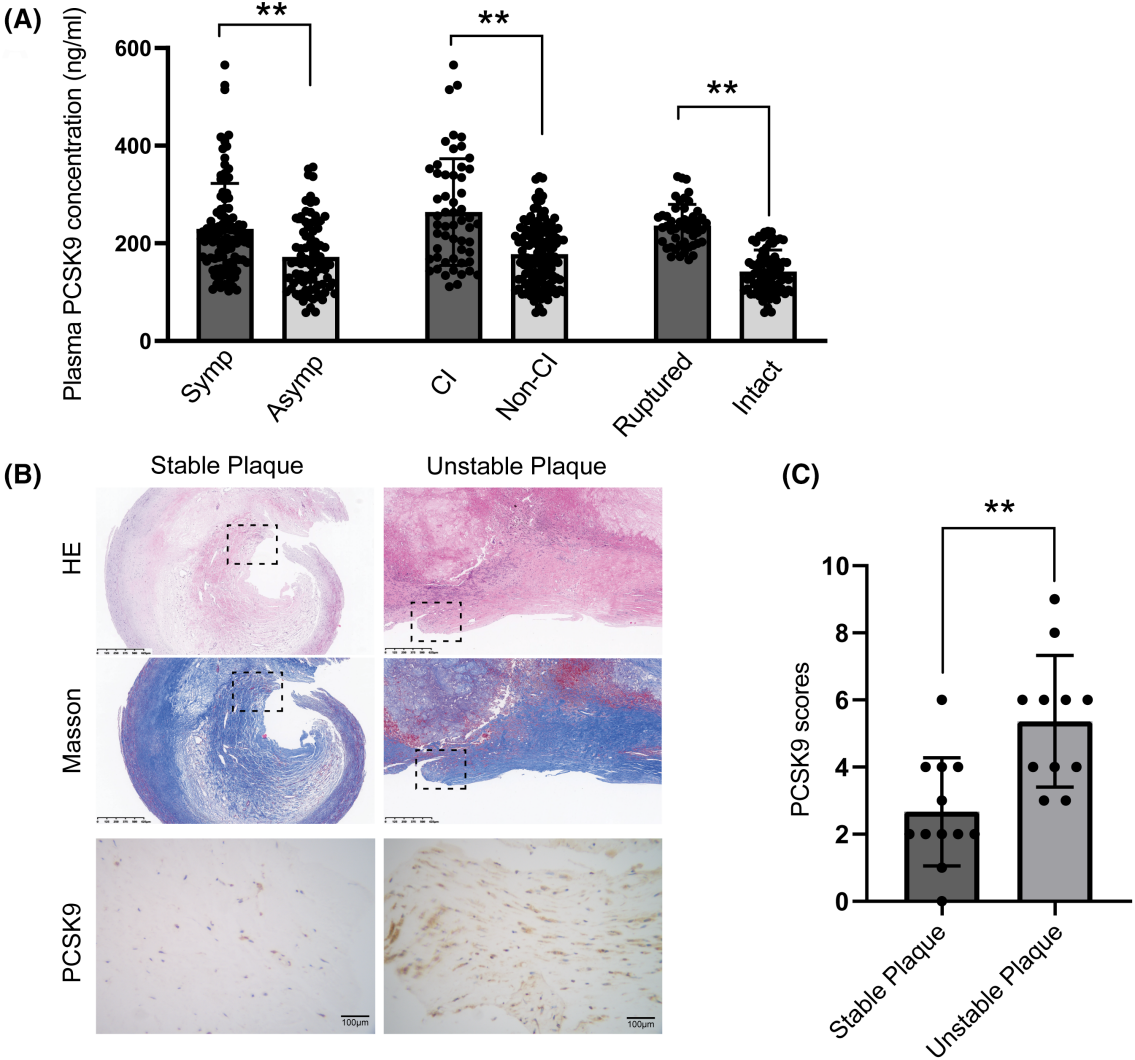
PCSK9 levels correlate with plaque vulnerability in patients with carotid artery stenosis. (A) Plasma PCSK9 levels in different subgroups of patients with carotid artery stenosis: symptomatic (Symp) versus asymptomatic (Asymp), cerebral infarction (CI) versus non cerebral infarction (Non‐CI), ruptured plaque (Ruptured) versus plaque with intact fibrous cap (Intact). (B) Hematoxylin–eosin (H&E) staining, Masson and immunohistochemistry for PCSK9 in representative stable plaques and unstable plaques. (C) Quantitative analysis of PCSK9 staining from enrolled stable and unstable carotid plaques. Bar charts show the mean with SD. **p < 0.01.

The unstable plaques are featured by ruptured plaque, which appears to have a higher likelihood of inducing cerebral infarction and corresponding clinical symptoms. In order to explore the relationship between PCSK9 and carotid plaque vulnerability, plasma PCSK9 in CAS patients were evaluated through ELISA. We analyzed a total of 183 patients according three classifications (symptomatic vs asymptomatic = 100: 83; cerebral infarction vs non‐cerebral infarction = 55: 128; ruptured plaque vs plaque with intact fibrous cap = 54: 129). The baseline clinical characteristics of these subjects are shown in Table 1 and Table S1. Circulating PCSK9 concentrations were significantly higher in symptomatic patients or cases diagnosed with cerebral infarction (Figure 1A, *p* < 0.01). In addition, the expression of PCSK9 was increased in CAS patients with ruptured plaque compared with those with intact fibrous cap (Figure 1A, *p* < 0.01). These results indicated that PCSK9 may be involved in plaque development in CAS. To further validate the relationship between PCSK9 expression and plaque vulnerability, 23 carotid artery plaques (12 stable plaques and 11 unstable plaques) were subjected to routine pathological examination (Figure 1B). Immunohistochemistry for PCSK9 was conducted and further confirmed the higher expression of PCSK9 in unstable plaques (Figure 1B,C). Overall, these results suggested that PCSK9 expression is associated with plaque vulnerability in CAS.

**TABLE 1:** Clinical characteristics of carotid atherosclerotic plaques included for histopathological analysis of PCSK9.

Characteristics	Number of patients (*n* = 183)
Age	64.35 ± 7.03
Male	151 (82.51)
BMI	24.77 (22.77–26.73)
Hypertension	127 (69.40)
Diabetes	57 (31.15)
Coronary heart disease	22 (12.02)
Hyperlipidemia	92 (50.27)
Smoking	
Never	82 (44.81)
Former	52 (28.42)
Current	49 (26.78)
Drinking	
Never	110 (60.11)
Former	24 (13.11)
Current	49 (26.78)
Laboratory test	
Triglycerides (mmol/L)	1.19 ± 0.68
Total Cholesterol (mmol/L)	3.35 ± 0.93
HDL Cholesterol (mmol/L)	1.03 ± 0.28
LDL Cholesterol (mmol/L)	1.80 ± 0.69
Apolipoprotein AI (g/L)	1.09 ± 0.24
Apolipoprotein B (g/L)	0.71 ± 0.23
PCSK9 concentration (ng/ml)	203.50 ± 88.91

*Note*: Categorical variables are presented as number (%). Continuous variables are presented as mean ± standard deviation or median (25th–75th percentile).

### 3.2 Increased PCSK9 expression leads to VSMCs' apoptosis

**FIGURE 2:**
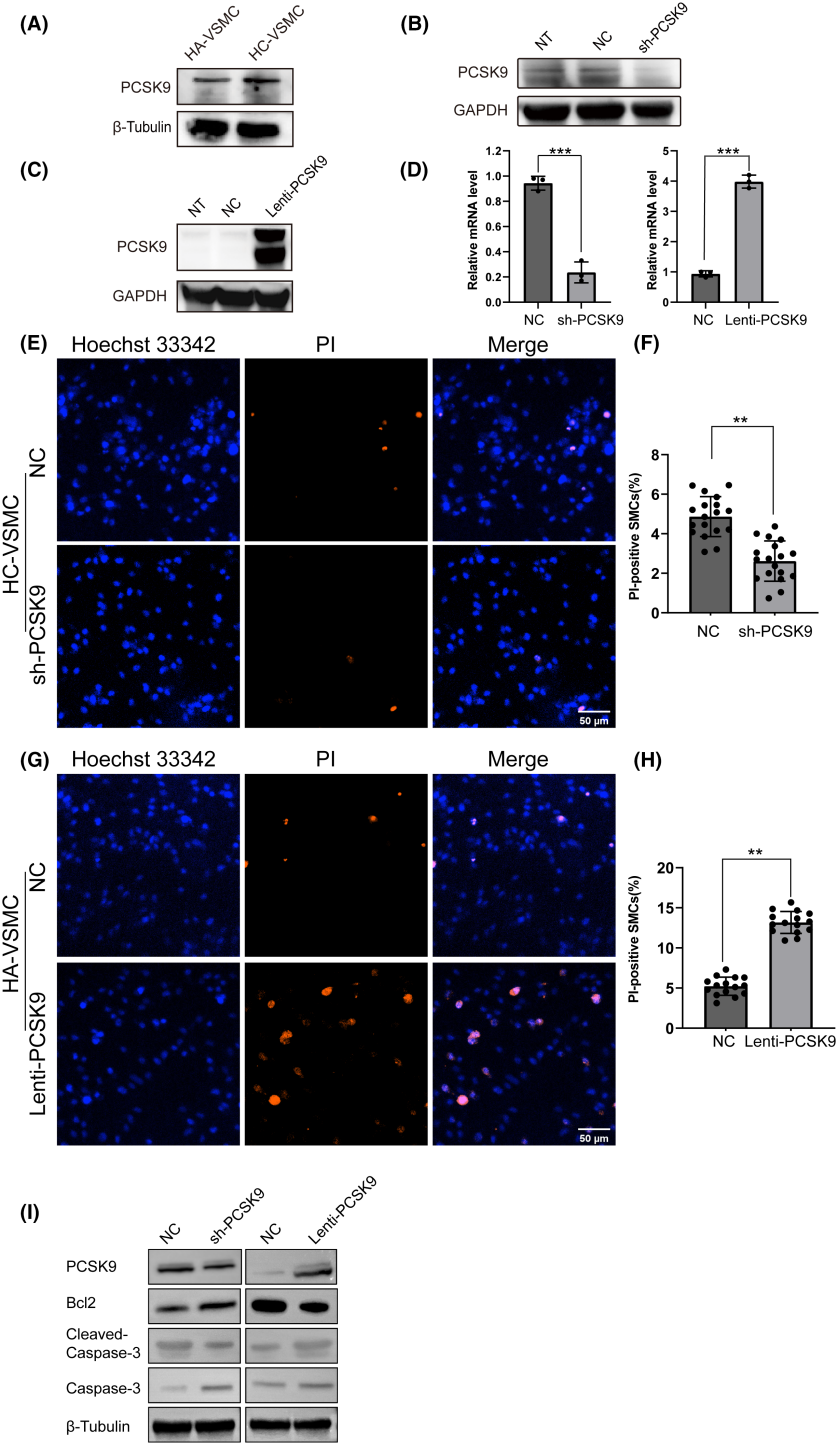
Increased PCSK9 expression leads to VSMCs' apoptosis. (A) PCSK9 expression in HC‐VSMCs and HA‐VSMCs compared by Western blot. (B) Knockdown and overexpression of PCSK9 was performed in HC‐VSMCs and HA‐VSMCs, respectively. The efficiency of knockdown and overexpression was examined by Western blot (B, C) and PCR (D). (E–H) Apoptosis were evaluated by PI staining (E, G) and quantitative analysis when PCSK9 was down‐ or up‐regulated in HC‐VSMCs (F) and HA‐VSMCs (H), respectively. Hoechst33342 (blue) labels cell nucleus and PI (red) labels apoptotic cells. (I) The level of PCSK9, Bcl2, Caspase‐3, Cleaved Caspase‐3 were analyzed by western blot, after PCSK9 was up‐ or down‐ regulated in HA‐VSMCs and HC‐VSMCs, respectively. Bar charts show the mean with SD. **p < 0.01; ***p < 0.001.

VSMCs have been recognized as the main cell type in construction and development of fibrous cap. In addition, the increased apoptosis of VSMCs in advanced‐stage plaques was one of the main causes of plaque rupture. We hypothesized that PCSK9 participated in behavioral regulation of VSMCs, since higher levels of PCSK9 in ruptured plaques compared with intact plaques is observed. Firstly, we measured the expression of PCSK9 in HA‐VSMCs and HC‐VSMCs, which demonstrated relatively higher expression of PCSK9 in HC‐VSMCs (Figure 2A). Then, knockdown and overexpression of PCSK9 by lentivirus was conducted in HC‐VSMCs and HA‐VSMCs, respectively. The efficiency of down‐ and up‐regulation of PCSK9 was confirmed at mRNA and protein level (Figure 2B–D). Propidium iodide (PI) staining was performed to explore direct evidence to support that PCSK9 regulates apoptosis in living VSMCs. The results showed that increased expression of PCSK9 facilitated apoptosis of HA‐VSMCs, while down‐regulation of PCSK9 protected HC‐VSMCs from apoptosis (Figure 2E–H).

There are three main signal cascades regulating cell apoptosis: intrinsic or mitochondrial pathways; external or death receptor (FAS and tumor necrosis factor) pathways, and endoplasmic reticulum stress‐mediated pathways.^20^ The results of Western blot showed that elevated PCSK9 expression in HA‐VSMCs increased the cleavage of mitochondrial apoptotic pathway indicator Caspase‐3 and decreased the expression of anti‐apoptotic protein Bcl2. Furthermore, knockdown of PCSK9 in HC‐VSMCs reduced the cleavage of Caspase‐3 and increased the expression of Bcl2 (Figure 2I). Therefore, PCSK9 may regulate the apoptosis of VSMCs via the mitochondrial‐related pathway.

### 3.3 PCSK9 may regulate mitochondrial morphology, ROS production, and membrane potential in VSMCs

**FIGURE 3:**
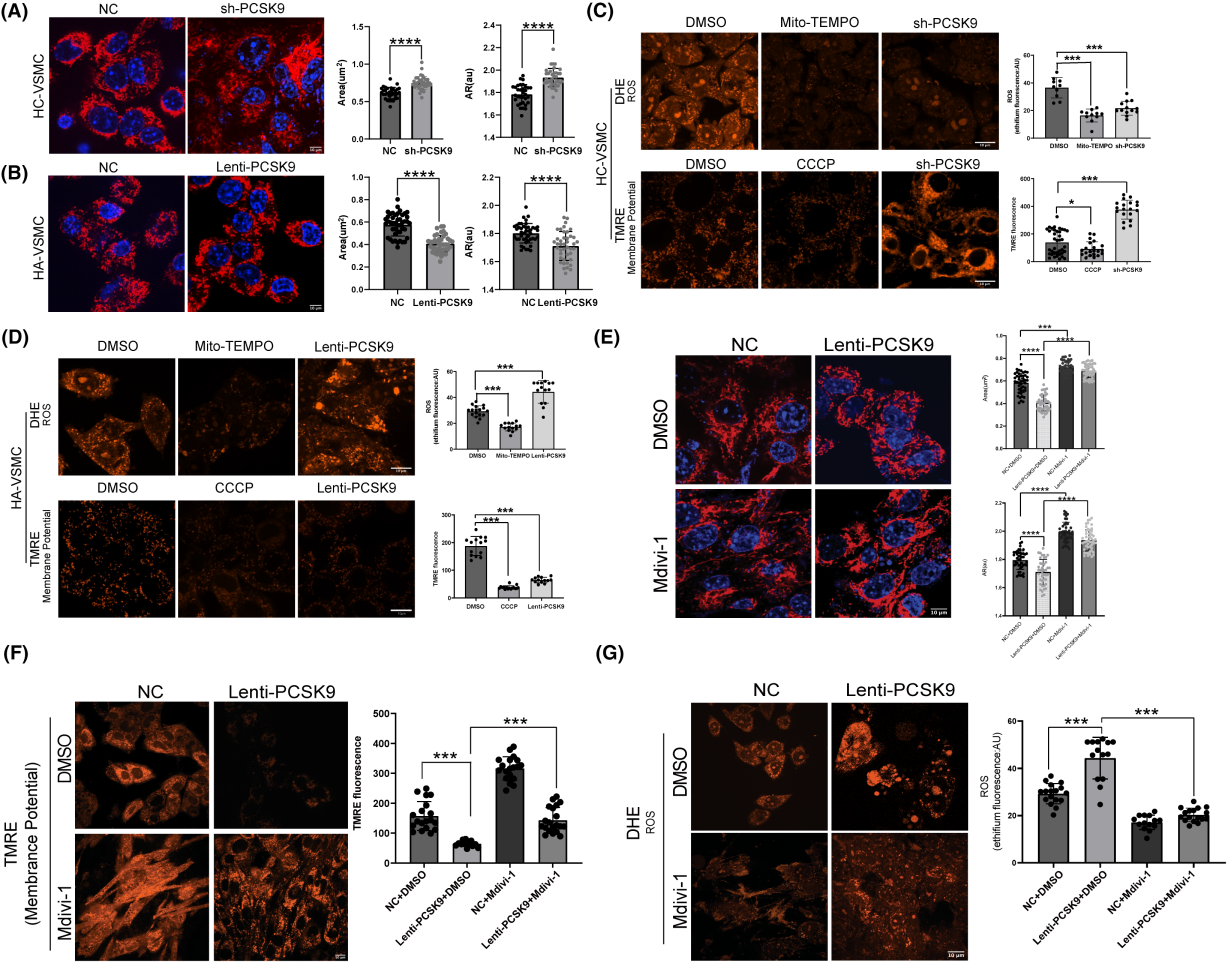
PCSK9 may regulate mitochondrial morphology, ROS production, and membrane potential in VSMCs. (A, B) Morphology changes of mitochondria and quantitative analysis when PCSK9 was down‐ or up‐regulated in HC‐VSMCs and HA‐VSMCs, respectively. Red color represents Mitotracker and blue color (Hoechst33342) represents cell nucleus. (C, D) Detection and quantitative analysis of mitochondrial ROS and mitochondrial membrane potential, when PCSK9 was down‐ or up‐regulated in HC‐VSMCs and HA‐VSMCs, respectively. (E) Morphological changes of mitochondria was compared by using Mitotracker (left panel) and analyzed (right panel) when activation of DRP1 was inhibited in Lenti‐PCSK9 HA‐VSMCs. (F, G) Detection and quantitative analysis of mitochondrial membrane potential and mitochondrial ROS, when activation of DRP1 was inhibited in Lenti‐PCSK9 HA‐VSMCs. AR, aspect ratio. Bar charts show the mean with SD. *p<0.05; ***p < 0.001; ****p < 0.0001.

To further examine the role of PCSK9 in mitochondria‐mediated apoptosis in VSMCs, we monitored mitochondria morphology using Mitotracker staining in live VSMCs. Inhibition of PCSK9 leads to elongated mitochondria with larger area and spatial ratio, while overexpression of PCSK9 resulted in the opposite results that reflected mitochondrial fragmentation were frequently observed (Figure 3A,B). We further checked the functional changes of mitochondria using the DHE probe indicating reactive oxygen species (ROS) and TMRE staining probing mitochondria membrane potential, respectively. Knockdown of PCSK9 reduced the production of ROS and increased the mitochondrial membrane potential, whereas overexpression of PCSK9 elevated the ROS level and downregulated the mitochondrial membrane potential (Figure 3C,D). Mitochondrial dysfunction is represented by dysregulated mitochondrial membrane potential and ROS, which promotes apoptosis. PCSK9 may participate in cell fate determination via modulating mitochondria function in VSMCs.

Mitochondria are membrane‐bound organelles, which are composed of the outer mitochondrial membrane, mitochondrial matrix, and inner mitochondrial membrane. In eukaryotic cells, the mitochondrial shape and function are maintained by balancing between fusion and fission which mainly regulated by dynamic related protein 1 (DRP1).^21^ We hypothesized that DRP1 may participate in regulation of PCSK9‐related mitochondrial morphology and function. In this line, Mdivi‐1, a specific antagonist of DRP‐1, partially restored the mitochondria shape and function caused by PCSK9 overexpression (Figure 3E–G). These findings preliminarily indicated that PCSK9 modulated the mitochondria morphology and function through the regulation of the DRP1.

### 3.4 PCSK9 promotes mitochondrial dysfunction by activating p38MAPK signaling

**FIGURE 4:**
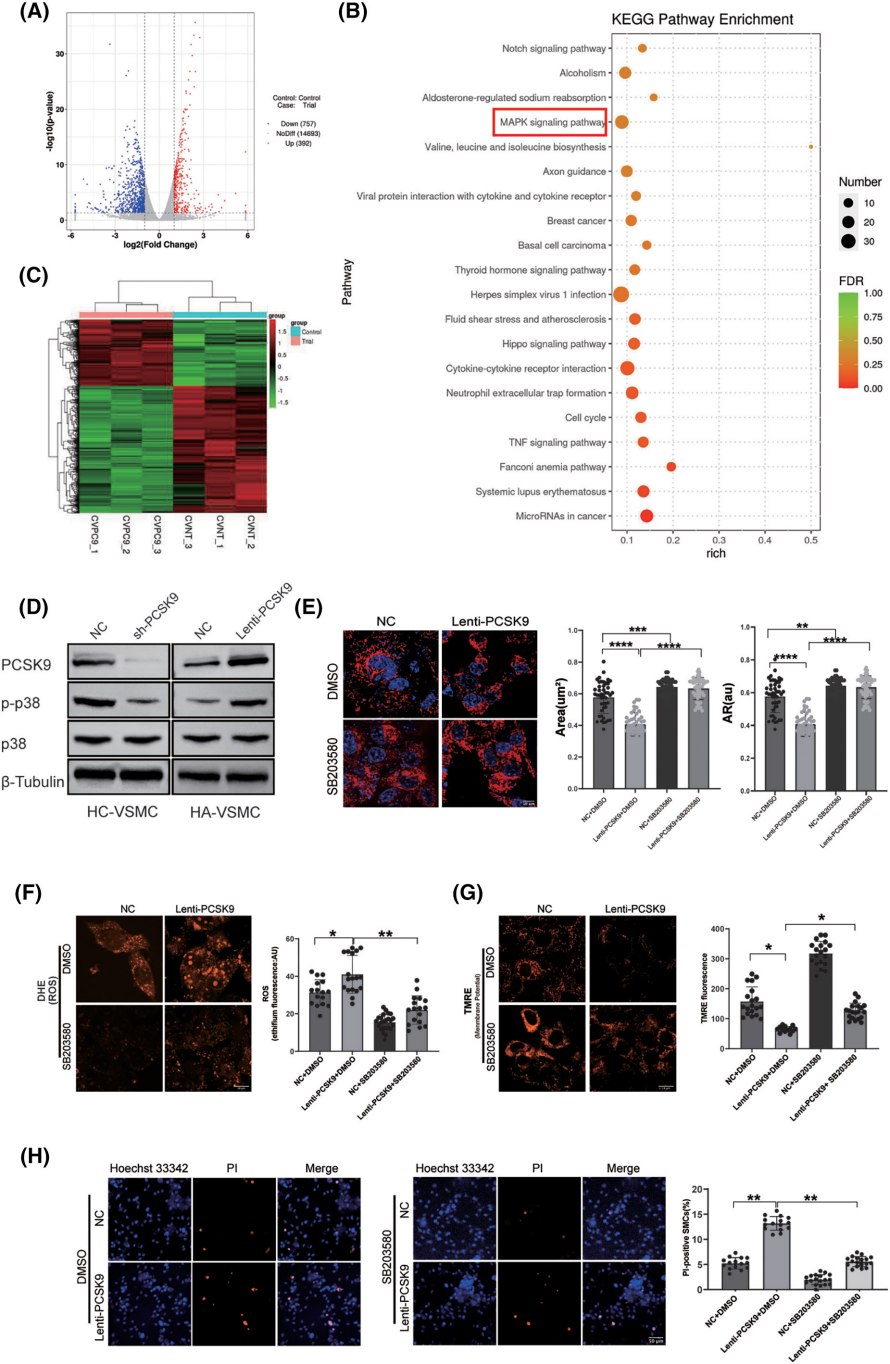
PCSK9 regulates morphology, function of mitochondrial and VSMCs' apoptosis through activation of p38MAPK. (A) Volcano plot of differentially expressed genes between NC and sh‐PCSK9 HC‐VSMCs. (B) KEGG pathway analysis of enriched differentially expressed genes. (C) Heatmap of differentially expressed gene clustering. Red representing highly‐expressed genes and green representing lower expressed genes. (D) The effect of PCSK9 level on activation of p38 was measured by western blot in HC‐VSMCs and HA‐VSMCs. (E) Morphological changes of mitochondria was compared by using Mitotracker (left panel) and quantitative analysis was performed (right panel) when activation of p38 was inhibited in Lenti‐PCSK9 HA‐VSMCs. (F, G) Detection and quantitative analysis of mitochondrial ROS (F) and mitochondrial membrane potential (G), when phosphorylation of p38 was inhibited in Lenti‐PCSK9 HA‐VSMCs. (H) PI staining and quantitative analysis before and after inhibition of p38 in NC and Lenti‐PCSK9 HA‐VSMCs. Hoechst33342 (blue) labels nucleus and PI (red) labels apoptotic cells. AR, aspect ratio. Bar charts show the mean with SD. *p < 0.05; **p < 0.01; ***p < 0.001; ****p < 0.0001.

To examine how PCSK9 regulates DRP1 and how it is linked to apoptosis regulation, control and sh‐PCSK9 HC‐VSMCs were subjected to RNA‐sequencing followed by bioinformatic analysis. Among 392 up‐regulated genes and 757 down‐regulated genes (Figure 4A–C), we noticed that mitogen‐activated protein kinases (MAPKs)‐associated genes were enriched among the signaling pathways (Figure 4B). According to a previous study, p38MAPK was recognized as a promotor of cell apoptosis.^22^ Thus, we tested the expression of p38MAPK in situation of PCSK9 knockdown and overexpression. The results showed decreased activation of p38MAPK in sh‐PCSK9 HC‐VSMCs and elevated phosphorylation of p38MAPK in Lenti‐PCSK9 HA‐VSMCs (Figure 4D). Based on the above phenomenon, SB203580, the inhibitor of p38MAPK phosphorylation was employed to examine the role of p38MAPK in mitochondrial function and regulation of apoptosis. We found that SB203580 partially restored the level of apoptosis, mitochondria shape and function caused by PCSK9 overexpression (Figure 4E–H). The above results suggested that the mediation of PCSK9 on mitochondria morphology and function may partially depend on phosphorylation regulation of p38MAPK.

### 3.5 The regulation of PCSK9 on mitochondrial dysfunction and apoptosis of VSMCs depends on 1‐149aa domain

**FIGURE 5:**
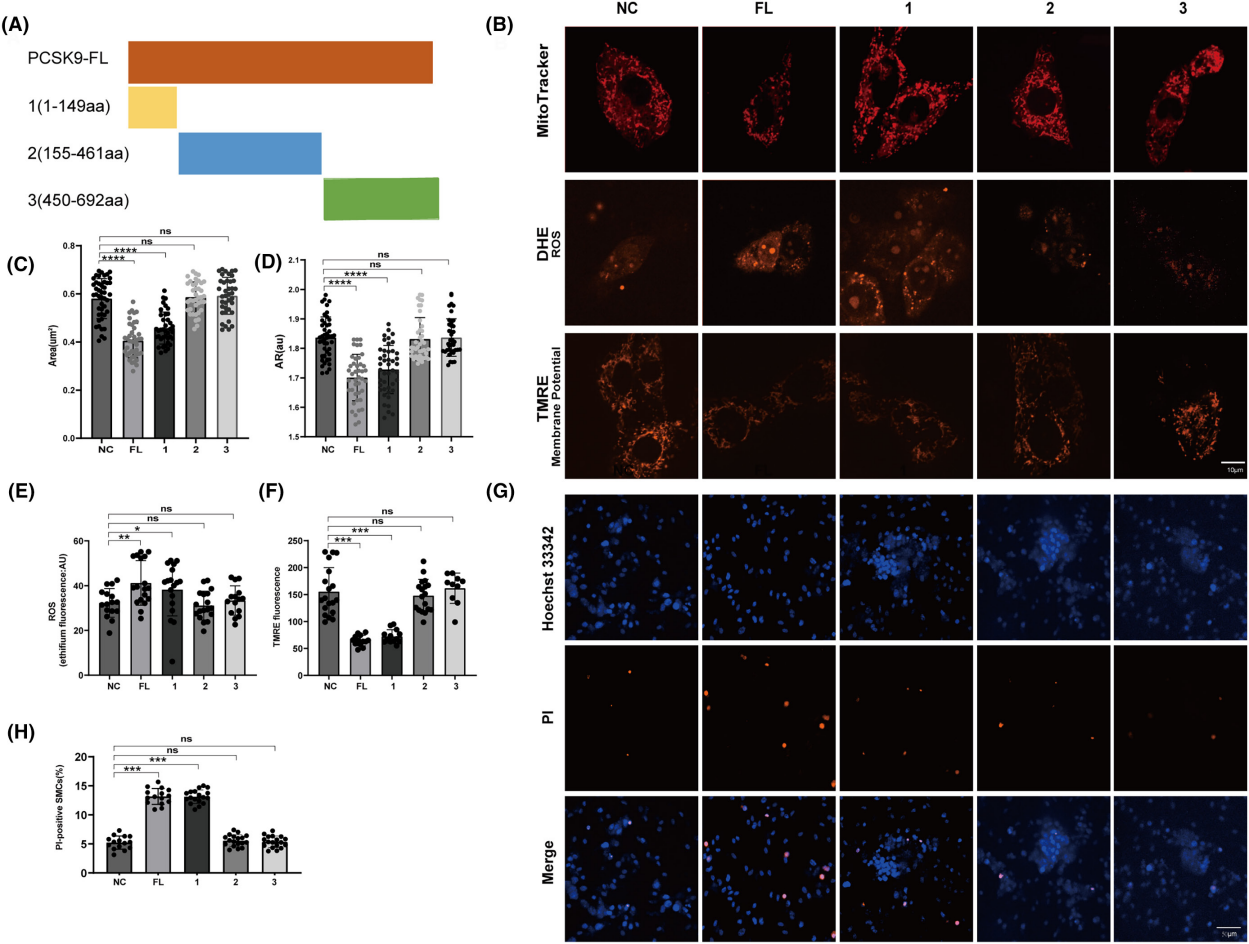
The regulation of PCSK9 on mitochondrial dysfunction and apoptosis of VSMCs depends on 1‐149aa domain. (A) Schematic diagrams for construction of PCSK9 short‐truncated variants (1, 1‐149aa; 2, 155‐461aa; 3, 450‐692aa). (B) Representative figures for mitochondrial morphology, ROS, and mitochondrial membrane potential of HA‐VSMCs transfected with different truncated plasmids of PCSK9. (C–F) Quantitative analysis of mitochondrial morphology (C, D), ROS (E), and mitochondrial membrane potential (F) of HA‐VSMCs transfected with different truncated plasmids of PCSK9. (G, H) PI staining and quantitative analysis after HA‐VSMCs were transfected with different truncated plasmids of PCSK9. Hoechst33342 (blue) labels nucleus and PI (red) labels apoptotic cells. AR, aspect ratio. Bar charts show the mean with SD. *p < 0.05; **p < 0.01; ***p < 0.001; ****p < 0.0001. ns, not significant.

According to protein domain information of PCSK9, we synthesized truncated plasmids to explore the essential domain of PCSK9‐regulated mitochondrial morphology, function and apoptosis of VSMCs (Figure 5A). The 1‐149aa, 155‐461aa and 450‐692aa was reserved when plasmid 1, 2, 3 was transfected in HA‐VSMCs, respectively. Then, we observed that the regulation of PCSK9 on mitochondrial morphology, function and VSMC apoptosis was partially abolished when 1‐149aa domain was absent, while similar phenomenon was not observed when the other two domains was truncated (Figure 5B–H). Thus, we speculated that the 1‐149aa of PCSK9 plays a key role in regulating mitochondrial morphology and apoptosis.

## 4 DISCUSSION

**FIGURE 6:**
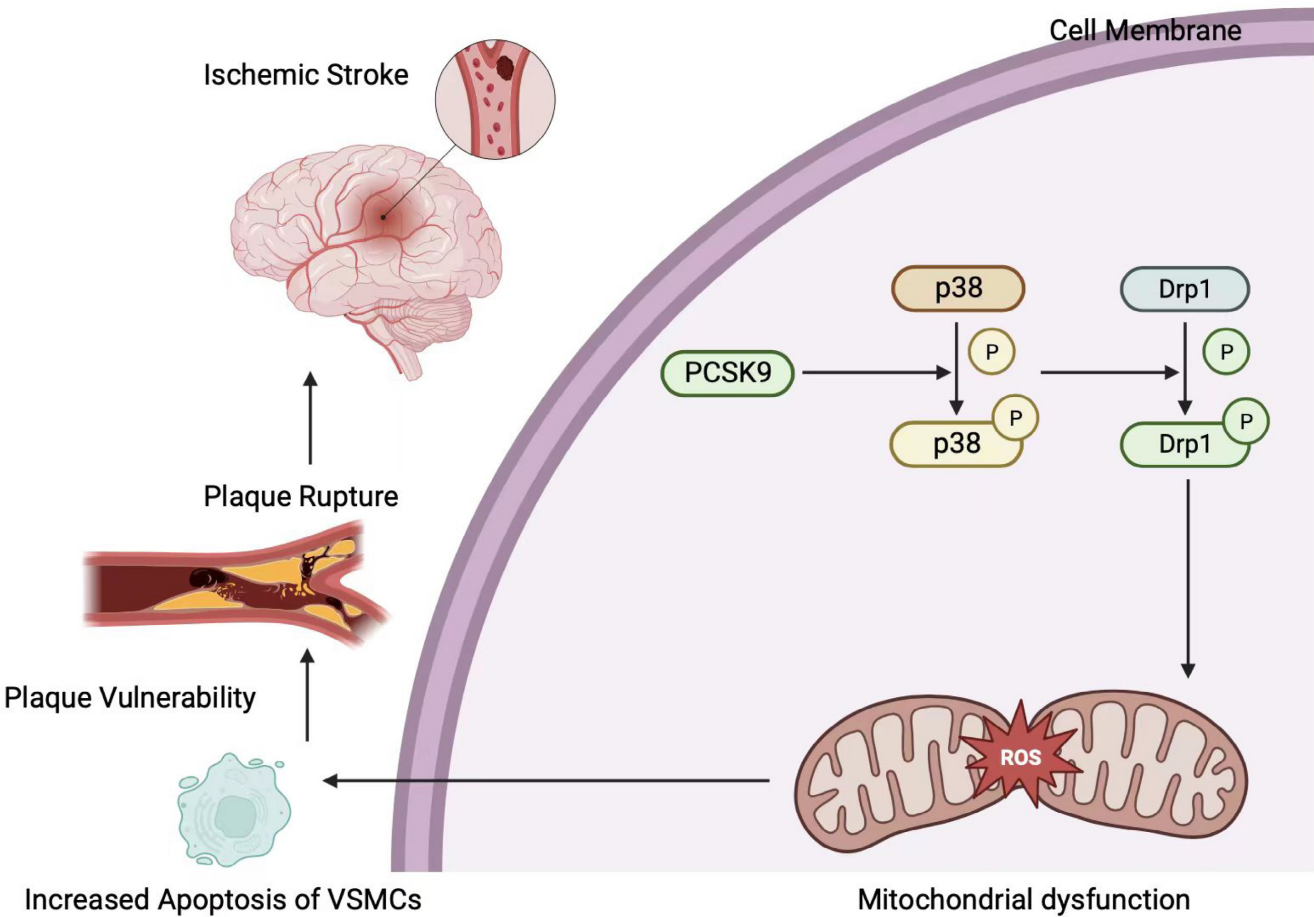
Schematic diagram of PCSK9‐mediated mitochondrial dysfunction and apoptosis of VSMCs. In human VSMCs, elevated PCSK9 promotes DRP1 activation through enhancing phosphorylation of p38, which resulted to mitochondrial morphological shift toward fission state, followed by abnormal increase of ROS and leads to mitochondrial dysfunction. The irregular function of mitochondria increases apoptosis of VSMCs, which contributes to formation of vulnerable carotid atherosclerotic plaques. Therefore, the plaques tends to rupture and results to thrombotic events, finally leads to the incidence of ischemic stroke.

This study explored the clinical significance of PCSK9 in carotid plaques, which suggested PCSK9 as a potential marker for stratification of high‐risk patients with carotid atherosclerotic stenosis (Figure 6). Up‐regulation of PCSK9 in human VSMCs increased mitochondrial‐associated apoptosis. At the subcellular level, we found that the morphology of mitochondria changed from fusion to fission state, which may relate to mitochondrial dysfunction. In this scenario, our RNA‐sequencing data from VSMCs and the employed biological assays indicated p38‐DRP1 as the potential underlying mechanism. This study aimed to determine the relationship between PCSK9 and carotid plaque vulnerability, and to explain the approach of PCSK9‐mediated apoptosis at subcellular level. Therefore, we may distill the rationale for application of PCSK9 inhibitor in management of unstable carotid plaques.

In fact, the clinical value of PCSK9 as a potential marker for subclinical carotid atherosclerosis has been investigated in a large cohort. Among 3708 European patients asymptomatic for cardiovascular diseases, PCSK9 plasma levels did not correlate with vascular damage and/or subclinical atherosclerosis of extracranial carotid arteries.^23^ Regarding to the progression of carotid atherosclerotic stenosis, plasma PCSK9 levels are associated with 10‐year progression of atherosclerosis as evaluated by total plaque area in 643 Asian patients.^24^ These studies provided a clue for exploring the further clinical value of PCSK9 in patients with carotid artery stenosis. In a cohort including 28 patients, the lipid content of carotid plaque was reduced by 17% after 6 months of PCSK9 inhibition with alirocumab.^25^ Furthermore, Shingai et al concluded that PCSK9 inhibitor may possibly stabilize carotid plaque and reduce perioperative complications of stenting based on a cohort of nine patients.^26^ Our study preliminarily determined the potential of plasma PCSK9 as an indicator of unstable carotid plaque (Table 1, Table S1). Our findings may enrich the evidence for the clinical application of PCSK9‐based diagnostics and prognosis prediction for patients with carotid atherosclerotic stenosis.

In early‐stage atherosclerosis, VSMCs migrate from the media to the intima and proliferate, wrap the necrotic core by secreting extracellular matrix, and inhibiting the rupture of the fibrous cap.^27^ However, VSMCs' apoptosis is abnormal in advanced plaques, which weakened fibrous cap and enlarged necrotic cores. VSMCs are the main source of phagocytes in plaques. Thus, the increased apoptosis of VSMCs directly leads to increased accumulation of necrotic components in plaques. However, there is currently no effective target to effectively intervene the excessive apoptosis of VSMCs in advanced plaques.^28^ Notably, among cells constructing the vessel wall, PCSK9 was only expressed in VSMCs.^29^ Therefore, our study was also performed in VSMCs. However, our results could not rule out whether PCSK9 in VSMCs may interact with other cell subtypes through secretion, thereby affecting plaque vulnerability.

To date, the mechanism of PCSK9‐mediated VSMCs' apoptosis in carotid plaque remains unclear. During plaque formation and development, mitochondria can affect cell metabolism and respiration, increase the production of ROS, and promote oxidative stress.^30^, ^31^ Analysis of plaque‐derived VSMCs demonstrates that mitochondrial respiration is dysregulated, the respiratory chain enzyme complex I expression is inhibited, and the mitophagy is enhanced.^32^ The above studies preliminarily indicate that mitochondrial dysfunction may be an important factor in the formation of unstable plaques. Inhibiting the expression of PCSK9 can significantly downregulate ROS in VSMCs.^33^ Our study found that the expression of PCSK9 regulated mitochondrial function and apoptosis of VSMCs from the perspective of mitochondrial dynamics. In fact, the balanced state of mitochondrial fusion and fission is closely related to mitochondrial oxidative metabolism, membrane potential and energy generation.^34^ Mechanistically, we found that DRP1, a key protein that regulates mitochondrial dynamics, may be a key downstream molecule of PCSK9. In human hepatocytes, DRP1 was reported to reduce PCSK9 secretion, drive mitochondrial fission by contributing to endoplasmic reticulum (ER) constriction of mitochondria.^35^ Along this line, we speculate that a feedback mechanism between PCSK9 and DRP1 may exist in VSMCs, which affects carotid plaque progression.

The current study has some limitations even though the potential value and novel mechanism of PCSK9‐initiated VSMCs' apoptosis was preliminarily clarified herein. Firstly, the sample size of our clinical cohort may not be sufficient to definitively characterize the relationship between PCSK9 and the subitems under characteristics of unstable plaque. Secondly, we identified the regulation of PCSK9 on mitochondrial dynamics and the related dysfunction and apoptosis. However, the detailed mechanism of PCSK9‐related mitochondrial dynamics of VSMCs remains to be determined. Future studies using advanced models may add more reliable evidence on subcellular approach of PCSK9‐assocaited carotid plaque vulnerability.

## 5 CONCLUSION

The expression of PCSK9 in carotid plaque tissue and plasma was positively related with vulnerability. Elevated PCSK9 in VSMCs led to excessive fission morphology and dysfunction of mitochondria, followed by increased apoptosis. The 1‐149aa segment of PCSK9 and the downstream p38‐DRP1 axis may be essential for this biological regulation. This study describes the relationship between PCSK9 and mitochondrial morphology and provides novel evidence for PCSK9 inhibitors in treatment of unstable carotid plaques.

## AUTHOR CONTRIBUTIONS

Liqun Jiao, Wenjing Li and Ran Xu designed and supervised all experiment of this study. Ran Xu, Tianhua Li and Jichang Luo performed histological and biological study. Tao Wang, Bin Yang, Yabing Wang, Yan Ma and Adam A Dmytriw analyzed and interpreted the patient data. Jinzhu Jia and Xiao Zhang contributed to data analysis. All authors involved in the writing of manuscript, read and approved the final manuscript.

## FUNDING INFORMATION

This study was supported by funding from Beijing Hospitals Authority's Ascent Plan (DFL20220702), the National Natural Science Foundation of China (Grant No. 82171303), and the Beijing Scientific and Technologic Project (Grant No. Z201100005520019, Grant No. Z201100005520020), Beijing Municipal Natural Science Foundation (Youth project, 7244353).

## CONFLICT OF INTEREST STATEMENT

The authors declare that they have no competing interests.

## Supporting information


Table S1.

## Data Availability

The datasets used and/or analyzed during the current study are available from the corresponding author on reasonable request.
