# Supplementary material for: PCSK9 increases vulnerability of carotid plaque by promoting mitochondrial dysfunction and apoptosis of vascular smooth muscle cells
Source: CNS Neurosci Ther. 2024 Feb 25;30(2):e14640. doi: 10.1111/cns.14640 (PMC10894644; doi:10.1111/cns.14640)
Supplement: Supplementary file 1 — Table S1. [file CNS-30-e14640-s001.docx]

**Table S1. Subgroup analysis for** **clinical characteristics of carotid plaques included for histopathological analysis of PCSK9**

| Characteristics | Symp (n=100) | Asymp (n=83) | P-value | CI (n=55) | Non-CI (n=128) | P-value | Ruptured (n=85) | Intact (n=98) | P-value |
| --- | --- | --- | --- | --- | --- | --- | --- | --- | --- |
| Age | 63.34±6.82 | 65.57±7.13 | **0.033** | 65.00 (59.00-70.00) | 66.00 (61.00-69.00) | 0.757 | 63.88±7.73 | 64.76±6.37 | 0.404 |
| Male | 81 (81.00) | 70 (84.34) | 0.696 | 45 (81.82) | 106 (82.81) | 0.836 | 67 (78.82) | 84 (85.71) | 0.246 |
| BMI | 24.85 (22.80-26.70) | 24.54 (22.77-26.73) | 0.660 | 25.14 (23.51-28.04) | 24.55 (22.65-26.33) | 0.121 | 24.65 (22.57-25.96) | 24.86 (23.08-27.06) | 0.185 |
| Hypertension | 69 (69.00) | 58 (69.88) | >0.999 | 39 (70.91) | 88 (68.75) | 0.862 | 52 (61.18) | 75 (76.53) | **0.036** |
| Diabetes | 32 (32.00) | 25 (30.12) | 0.873 | 19 (34.55) | 38 (29.69) | 0.602 | 27 (31.76) | 30 (30.61) | 0.874 |
| Coronary heart disease | 8 (8.00) | 14 (16.87) | 0.073 | 5 (9.09) | 17 (13.28) | 0.62 | 9 (10.59) | 13 (13.27) | 0.653 |
| Hyperlipidemia | 47 (47.00) | 45 (54.22) | 0.374 | 23 (41.82) | 69 (53.91) | 0.149 | 39 (45.88) | 53 (54.08) | 0.301 |
| Smoking |  |  |  |  |  |  |  |  |  |
| Never | 46 (46.00) | 36 (43.37) |  | 22 (40.00) | 60 (46.88) |  | 39 (45.88) | 43 (43.88) |  |
| Former | 27 (27.00) | 25 (30.12) | 0.892 | 16 (29.09) | 36 (28.13) | 0.631 | 24 (28.24) | 28 (28.57) | 0.956 |
| Current | 27 (27.00) | 22 (26.51) |  | 17 (30.91) | 32 (25.00) |  | 22 (25.88) | 27 (27.55) |  |
| Drinking |  |  |  |  |  |  |  |  |  |
| Never | 62 (62.00) | 48 (57.83) |  | 29 (52.73) | 81 (63.28) |  | 54 (63.53) | 56 (57.14) |  |
| Former | 13 (13.00) | 11 (13.25) | 0.822 | 8 (14.55) | 16 (12.50) | 0.392 | 10 (11.76) | 14 (14.29) | 0.676 |
| Current | 25 (25.00) | 24 (28.92) |  | 18 (32.73) | 31 (24.22) |  | 21 (24.71) | 28 (28.57) |  |
| Laboratory test |  |  |  |  |  |  |  |  |  |
| Triglycerides (mmol/L) | 1.15±0.59 | 1.24±0.77 | 0.406 | 1.39±0.75 | 1.11±0.63 | **0.01** | 1.30 (0.81-1.78) | 0.93 (0.63-1.24) | **P <0.001** |
| Total Cholesterol (mmol/L) | 3.19 (2.68-3.66) | 3.28 (2.76-3.71) | 0.31 | 3.47±0.90 | 3.30±0.94 | 0.278 | 3.48±1.06 | 3.25±0.79 | 0.09 |
| HDL Cholesterol (mmol/L) | 1.00 (0.83-1.26) | 0.97 (0.86-1.13) | 0.348 | 1.00 (0.82-1.16) | 0.99 (0.86-1.25) | 0.384 | 1.01 (0.85-1.19) | 0.94 (0.83-1.14) | 0.245 |
| LDL Cholesterol (mmol/L) | 1.72±0.55 | 1.90±0.82 | 0.082 | 1.79 (1.33-2.14) | 1.69 (1.31-2.01) | 0.346 | 1.74 (1.40-2.08) | 1.68 (1.30-2.04) | 0.425 |
| Apolipoprotein AI (g/L) | 1.10 (0.93-1.29) | 1.05 (0.92-1.19) | 0.175 | 1.11 (0.98-1.32) | 1.04 (0.89-1.21) | 0.09 | 1.17 (1.04-1.32) | 1.00 (0.88-1.14) | **P<0.001** |
| Apolipoprotein B (g/L) | 0.69±0.24 | 0.73±0.23 | 0.319 | 0.74±0.55-0.83 | 0.66±0.55-0.77 | 0.544 | 0.73±0.26 | 0.68±0.21 | 0.126 |
| PCSK9 concentration (ng/ml) | 229.70±93.12 | 172.00±72.40 | **P<0.001** | 239.80 (168.90-343.30) | 172.40 (124.90-223.60) | **P<0.001** | 248.70 (218.00-303.60) | 143.40 (111.70-171.40) | **P<0.001** |

Symp, Symptomatic; Asym, Asyptomatic; CI, cerebral infarction. HDL, high density lipoprotein; LDL, low density lipoprotein.
